# Adenosine A_3_ receptor antagonists as anti‐tumor treatment in human prostate cancer: an *in vitro* study

**DOI:** 10.1002/2211-5463.70024

**Published:** 2025-04-03

**Authors:** Maria Beatrice Morelli, Andrea Spinaci, Cui Chang, Rosaria Volpini, Catia Lambertucci, Matteo Landriscina, Vincenza Conteduca, Consuelo Amantini, Cristina Aguzzi, Laura Zeppa, Martina Giangrossi, Laura Soverchia, Matteo Santoni, Massimo Nabissi, Giorgio Santoni, Carlo Polidori

**Affiliations:** ^1^ Experimental Medicine Unit School of Pharmacy, University of Camerino Italy; ^2^ Medicinal Chemistry Unit School of Pharmacy, University of Camerino Italy; ^3^ Unit of Medical Oncology and Biomolecular Therapy, Department of Medical and Surgical Sciences – Ospedali Riuniti University of Foggia Foggia Italy; ^4^ School of Bioscience and Veterinary Medicine University of Camerino Italy; ^5^ Oncology Unit Macerata Hospital Macerata Italy

**Keywords:** adenosine, adenosine receptor antagonists, chemotherapy, PC3 cell line, prostate cancer

## Abstract

Prostate cancer (PCa) is one of the most common cancers in men, and for patients with PCa that cannot be surgically resected or treated, androgen suppression therapy often results in significant adverse effects. Recent studies have shown that A3 adenosine receptors (A_3_ARs) are overexpressed in prostate cancer (PCa), and several A_3_AR agonists and antagonists have been investigated as potential anticancer drugs. In this study, we investigated the potential therapeutic effects of the A_3_AR antagonists AR 292 and AR 357 in human PCa cell lines. LNCaP, DU‐145, and PC3 cell lines were treated with AR 292 and AR 357 compounds, and their cytotoxic effects were determined using viability assays, flow cytometry, and western blotting. Moreover, the drug transporter gene profile was evaluated using RT‐PCR in untreated and A_3_AR antagonist‐treated PCa cells. Both AR 292 and AR 357 showed antiproliferative effects with significant cell cycle arrest and induced DNA damage leading to cell death. AR 292 and especially AR 357 modulated the expression of drug transporter genes involved in chemoresistance, ferroptosis, and the hypoxia response. Ferroptosis was induced in DU‐145 cells treated with both compounds as well as in PC3 cells treated with AR 357. However, the treatment of PC3 cells with AR 292 and the treatment of LNCaP cells with both AR 292 and AR 357 resulted in necrotic cell death. In conclusion, our study showed that A_3_AR ligands exert anticancer effects via different mechanisms on PCa cell lines through the activation of multiple molecular pathways.

## Graphical abstract

The A3 adenosine receptors (A_3_ARs) are overexpressed in prostate cancer. AR 292 and AR 357, as A_3_AR antagonists, are capable of blocking proliferation, modulating the expression of drug transporter genes involved in chemoresistance, ferroptosis, and the hypoxia response, and inducing cell death.

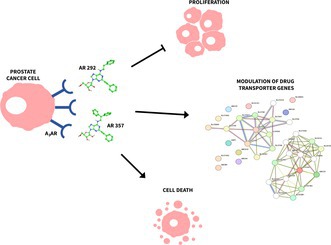

**Abbreviations**
A_3_Ars: A3 adenosine receptors
ABC: adenosine triphosphate‐binding cassette
Ado: adenosine
ADT: androgen deprivation therapy
APQ: aquaporin
AR 292: 2‐phenylethynyl‐*N* ^6^‐(2‐phenethyl)adenosine
AR 357: *N* ^6^‐(2,2‐diphenylethyl)‐2‐phenylethynyladenosine
CFSE: carboxyfluorescein succinimidyl ester
Cl‐IB‐MECA: 2‐chloro‐*N* ^6^‐(3‐iodobenzyl)Ado‐5’‐N‐methylcarboxamide
DCFDA: 2′,7′‐dichlorofluorescein
DMSO: dimethyl sulfoxide
FBS: fetal bovine serum
FDA: Food and Drug Administration
MASH: metabolic dysfunction‐associated steatohepatitis
NADPH: nicotinamide adenine dinucleotide phosphate hydrogen
NF‐kB: nuclear factor kappa B
Pca: prostate cancer
PPIs: protein–protein interaction
PrECs: primary prostate epithelial cells
SLC: solute carrier transporter
SRB: sulforhodamine B
STRING: Search Tool for Retrieval of Interacting Genes
Wnt: wingless‐related integration site

video

Prostate cancer (PCa) is one of the most common cancers in men and the second leading cause of cancer‐related deaths worldwide [1]. If detected early, PCa can be treated by surgery and/or radiation. However, if allowed to progress, the disease becomes incurable. Patients with PCa who cannot be surgically resected or treated with local radiation are commonly administered androgen deprivation therapy (ADT) as the basis of systemic treatment [2].

Adenosine (Ado) exerts its functions by binding to four G‐protein‐coupled receptors named A_1_, A_2A_, A_2B_, and A_3_. A_3_ adenosine receptors (A_3_ARs) are expressed in normal and neoplastic cells. Epithelial cells, chondrocytes, osteoblasts, mast cells, eosinophils, neutrophils, monocytes, macrophages, dendritic cells, and lymphocytes express A_3_ARs [3, 4]. Overexpression of A_3_ARs has been detected in melanoma, breast, colon, prostate, liver, pancreatic, and lung adenocarcinoma, lymphoma, and glioblastoma [3, 5]. It is clear that A_3_AR plays an important role in cancer; however, its function is still debated because it mediates both pro‐tumoral and anti‐tumoral effects. It can promote cell proliferation, angiogenesis, and migration/invasion, reduce cell proliferation and migration, and trigger apoptosis [6, 7].

Several A_3_AR agonists and antagonists have been investigated as potential anticancer drugs. Among them, the A_3_AR agonists IB‐MECA, Cl‐IB‐MECA, and thio‐Cl‐IB‐MECA can induce apoptotic cell death in several types of cancer cells [7]. Regarding PCa, Ado and A_3_ARs have been reported in PCa patients and cell lines [8, 9]. Activation of A_3_AR by IB‐MECA deregulates the Wnt and NF‐kB signaling pathways, resulting in the inhibition of PC3 cell growth [10]. Moreover, *in vitro* and *in vivo* activation of A_3_AR by IB‐MECA in rat AT6.1 PCa and human PC3‐MM cells reduced NADPH oxidase activity and cancer cell invasion [8]. In *in vitro* experiments, it inhibits androgen‐dependent and androgen‐independent PCa cell proliferation by inducing G1 cell cycle arrest through the p53/Cdk4/cyclin D pathways. Moreover, through caspase‐3 activation and Bcl‐2 downregulation, Ado induces mitochondrial apoptosis [9]. Furthermore, Cl‐IB‐MECA was found to induce reactive oxygen species (ROS)‐dependent autophagic cell death in PC3 cells [11]. This A_3_AR agonist, also called Namodenoson or CF 102, has been evaluated in clinical trials by Can‐Fite BioPharma Ltd. (Israel) for different tumor types and recently obtained permission from the US Food and Drug Administration (FDA) for a phase II clinical trial to treat metabolic dysfunction‐associated steatohepatitis (MASH) [12, 13]. For these reasons, it has become the reference compound for the anticancer biological evaluation of A_3_AR ligands.

A_3_AR antagonists may also be useful in treating PCa. We recently reported the synthesis and anti‐tumor activity of A_3_AR ligands represented by 2‐*N* ^6^‐disubstituted Ado derivatives. Among them, AR 292 and AR 357 (compounds 11 and 12) exhibited the best activity in the PC3 cell line [14]. Functional studies demonstrated their A_3_AR antagonistic behavior, and their anti‐tumor activity was higher than that of Cl‐IB‐MECA; however, the molecular mechanisms underlying their anticancer effects are currently unknown. Thus, the aim of this study was to evaluate the molecular mechanisms involved in the potential therapeutic anticancer effects of the already reported AR 292 and AR 357 A_3_AR antagonists in androgen‐dependent LNCaP and androgen‐independent DU‐145 and PC3 cell lines. Moreover, since there was a strong association between altered expression of ATP‐binding cassette (ABC) and solute carrier transporter (SLC) drug efflux, aquaporin (APQ) drug transporter, and ATP‐dependent drug efflux pumps, and tumor progression and increased resistance to chemotherapy, we also evaluated the expression of a drug transporter profile in neoplastic cells compared to that in normal prostate epithelial cells and the effect of AR 292 or AR 357 A_3_AR antagonists in PCa cell lines.

## Material and methods

### Cell lines

Immortalized primary prostate epithelial cells (PrECs), provided by Abmgood (Richmond, Canada), were isolated from healthy human prostate tissue and cultured in PriCoat™ T25 Flasks in Prigrow X (Abmgood).

The PCa cell lines LNCaP (RRID:CVCL_0395), DU‐145 (RRID:CVCL_0105), and PC3 (RRID:CVCL_0035) were purchased from Leibniz Institute DSMZ (Braunschweig, Germany). LNCaP represents the early stages of PCa because it exhibits androgen‐sensitive growth and is wild type for *TP53*. Instead, DU‐145 and PC3 represent advanced diseases because they are androgen‐insensitive and contain nonfunctional p53. LNCaP cells were maintained in RPMI‐1640 (Euroclone, Milan, Italy) supplemented with 20% heat‐inactivated fetal bovine serum (FBS), 100 IU·mL^−1^ penicillin, and 100 μg·mL^−1^ streptomycin. DU‐145 cells were maintained in RPMI‐1640 medium supplemented with 10% FBS, 100 IU·mL^−1^ penicillin, and 100 μg·mL^−1^ streptomycin. PC3 cells were maintained in F‐12K Nut MIX (Gibco, Milan, Italy), supplemented with 10% FBS, 100 IU·mL^−1^ penicillin, and 100 μg·mL^−1^ streptomycin. All the cell lines were grown at 37 °C in a humidified atmosphere containing 5% CO_2_.

### Compounds

2‐Chloro‐*N* ^6^‐(3‐iodobenzyl)Ado‐5’‐N‐methylcarboxamide (Cl‐IB‐MECA) (Tocris Bioscience, Bristol, UK) was dissolved in dimethyl sulfoxide (DMSO). AR 357 (*N* ^6^‐(2,2‐diphenylethyl)‐2‐phenylethynylAdo) and AR 292 (2‐phenylethynyl‐*N* ^6^‐(2‐phenethyl)adenosine) were dissolved in DMSO to obtain 50 mm stock solutions and diluted in medium on the same day of the experiment to adjust the final concentration of DMSO so as not to exceed 0.5% (v/v) in cell cultures. Sorafenib (Sigma Aldrich, Milan, Italy) was dissolved in DMSO to obtain a 2 mg·mL^−1^ stock solution. Liproxstatin‐1 (Lipro‐1, Sigma Aldrich) was dissolved in DMSO to obtain a 10 mg·mL^−1^ stock solution.

### Cell growth assay

PCa cells (4 × 10^4^ PCa cells·mL^−1^) were plated in a 96‐well plate. The day after, Cl‐IB‐MECA, AR 292, AR 357 (1–100 μm) or vehicle was added for 48 h. Cells were fixed with cold trichloroacetic acid (TCA) and incubated for 1 h at 4 °C. A solution of 100 μm sulforhodamine B (SRB) at 0.4% (w/v) in 1% acetic acid was added to each well, and the plates were incubated for 10 min at room temperature. After staining, the plates were air‐dried. Bound stain was solubilized by adding 100 μL of Trizma base. The absorbance at 560 nm was read on a SpectraMax iD3 multi‐mode microplate reader and analyzed by SoftMax Pro 7 Software version 7.2.0 (Molecular Device, Munchen, Germany). The absorbance at *t* = 0 was compared with the absorbance at the end of the experiment to determine cell growth in treated cells compared with control cells.

PrECs·mL^−1^ (5 × 10^4^) were plated in a 96‐well plate coated with Applied Cell Extracellular Matrix (Abmgood) and treated with Cl‐IB‐MECA, AR 292, AR 357 (1–100 μm), or vehicle for 48 h. The samples were incubated for 3 h with 0.8 mg·mL^−1^ of 3‐(4,5‐dimethylthiazol‐2‐yl)‐2,5‐diphenyltetrazolium bromide (MTT) and read on a spectrophotometer to measure the absorbance of the formazan crystals dissolved in 100 μL of DMSO per well. Four replicates were used for each treatment.

### Cell death assay

PCa cells (2.4 × 10^4^/well) treated with AR 292 or AR 357 for 48 h were incubated with 5 μL Annexin V‐FITC (Thermo Fisher Scientific, Rome, Italy) or 20 μg·mL^−1^ propidium iodide (PI; Sigma Aldrich) for 10 min at room temperature. After washing, fluorescence was analyzed by BD Accuri C6 Plus flow cytometer and BD Accuri C6 Plus Software version 1.0.34.1 (BD Biosciences, Milan, Italy), measuring the fluorescence emission on FL‐1 (530 BP) for Annexin V‐FITC and FL‐2 (585 BP) for PI.

### Cell cycle assay

PCa cells (1.2 × 10^5^ cells·well^−1^) seeded in 6‐well plates were exposed to AR 292 and AR 357 for 48 h. Next, the harvested cells were fixed in cold 70% ethanol, treated for 30 min at 37 °C with 100 μg·mL^−1^ ribonuclease A solution, stained for 30 min at room temperature with PI 20 μg·mL^−1^, and analyzed using a BD Accuri C6 Plus flow cytometer and its software, measuring the fluorescence emission on FL2‐A (585 BP).

### Cell proliferation assay

The cells were labeled using the CellTrace™ Cell Proliferation kit (Thermo Fisher Scientific) according to the manufacturer's instructions. Briefly, cells were washed with PBS and resuspended at 10^6^ cells·mL^−1^ in 1–2 μm carboxyfluorescein succinimidyl ester (CFSE) for 20 min at 37 °C. Five volumes of cell culture medium were then added, and the cell mixtures were allowed to rest for 5 min to remove free dye. The labeled cells were centrifuged, seeded at 4 × 10^4^ cells·mL^−1^, and cultured for 48 h in a culture medium containing AR 292, AR 357, or vehicle. At the end of treatment, fluorescence was evaluated using a BD Accuri C6 Plus flow cytometer and its software by measuring the fluorescence emission on FL‐1 (530 BP).

### ROS production

Oxidative stress levels in PCa cells were assessed by staining with 20 the fluorescent probe 2′,7′‐dichlorofluorescein (DCFDA). Cells were seeded into 6‐well culture plates (2.4 × 10^4^ cells·well^−1^) and exposed to AR 292 and AR 357 for 24 and 48 h. Following the incubation periods, the suspended cells were treated with the DCFDA fluorescent probe at 37 °C for 20 min. ROS production was evaluated using a BD Accuri C6 Plus flow cytometer and its software, by measuring the fluorescence emission on FL‐1 (530 BP).

### Determination of lipid ROS

Intracellular lipid ROS levels were measured using the fluorescent probe BODIPY 581/591 C11 according to the manufacturer's instructions (Thermo Fisher Scientific). In brief, PCa cells were first seeded in 24‐well plates at a density of 2.4 × 10^5^4^ cells per well. The cells were treated with AR 292 and AR 357 for 48 h, washed, and treated with 10 μm C11 BODIPY for 30 min at 37 °C in the dark. After incubation, the cells were washed with PBS and analyzed using a BD Accuri C6 Plus flow cytometer and its software. C11‐bodipy was excited at 561 nm to show the red fluorescence intensity associated with the unoxidized dye and excited at 488 nm to show the green fluorescence intensity associated with the oxidized dye.

### Western blot

Lysates from PCa cells were extracted using lysis buffer containing a protease‐inhibitor cocktail (EuroClone). Proteins were separated on 7%–14% SDS/polyacrylamide gels using a Mini‐PROTEAN Tetra Cell system (Bio‐Rad, Milan, Italy). Protein transfer to a nitrocellulose membrane was performed using a Mini Trans‐Blot Turbo RTA system (Bio‐Rad). Non‐specific binding sites were blocked with low‐fat dry milk or BSA in PBS containing 0.1% Tween 20. Membranes were incubated with anti‐Bcl‐xl (sc‐8392, 1:1000; Santa Cruz Biotechnology, Heidelberg, Germany), anti‐phospho H2A.X (#9718, 1:1000; Cell Signaling Technology, Milan, Italy), anti‐pERK (#9106, 1:1000; Cell Signaling Technology), anti‐ERK (#9102, 1:1000; Cell Signaling Technology), anti‐PARP‐1 (sc‐8007, 1:500; Santa Cruz Biotechnology), anti‐Caspase‐3 (#14220, 1:1000; Cell Signaling Technology), anti‐HIF‐1α (sc‐13 515, 1:500; Santa Cruz Biotechnology), anti‐β‐actin (sc‐47 778, 1:1000; Santa Cruz Biotechnology), or anti‐GAPDH (sc‐47 724, 1:1000; Santa Cruz Biotechnology) antibodies overnight at 4 °C, followed by incubation with HRP‐conjugated secondary antibodies (anti‐rabbit Ab, 1:5000; anti‐mouse, 1:2000; Cell Signaling Technology). Analysis was performed using LiteAblot PLUS or Turbo kits (EuroClone), ChemiDoc™ XRS+ with Image Lab™ Software version 6.1.0 (Bio‐Rad). GAPDH and β‐actin were used as loading controls. Representative results from three independent experiments are shown.

### RT–PCR profiler Array

Total RNA was extracted and reverse‐transcribed using the RNeasy Mini Kit (Qiagen, Milan, Italy) and RT^2^ First Strand Kit (Qiagen). qRT–PCR reaction was performed using the IQ5 Multicolor Real‐time PCR detection system (Bio‐Rad), the RT^2^ SYBR Green qPCR Mastermix (Qiagen) and the RT^2^ Profiler™ PCR Array Human Drug Transporters (Qiagen) according to the manufacturer's instructions. IQ5 Optical System Software version 2.1 is the real‐time PCR analysis software used. The ∆∆Ct‐based fold change and statistical significance analysis was performed using the Integrated Web‐based Software Package for the PCR Array System at the GeneGlobe Data Analysis Center on the Qiagen website (https://geneglobe.qiagen.com/us/analyze). Fold change (FC) cut‐offs of >2 and significance *P*‐values of <0.05 were considered statistically significant.

### Protein–protein interaction (PPI) network analysis

The Search Tool for Retrieval of Interacting Genes (STRING) (https://string‐db.org) database, based on known and predicted PPIs, was used to identify potential interactions between the markers [15]. Text mining, experiments, databases, co‐expression, species limited to “Homo sapiens,” and an interaction score >0.7 were considered active interaction sources and applied to construct the PPI networks. The PPI network was visualized using Cytoscape software version 3.9.1 (https://cytoscape.org/) [16]. In networks, proteins are schematized as nodes and interactions as edges.

### Statistical analysis

The statistical significance was determined by Student's *t*‐test and one‐way ANOVA with Tukey's multiple comparison test, *P < 0.05; **P < 0.01; ***P < 0.001; ****P < 0.0001. The statistical analysis was performed using GraphPad Prism 9.0.1 software (GraphPad Software, San Diego, CA, USA).

## Results

### A_3_AR involvement in PCa cell growth

**Fig. 1:**
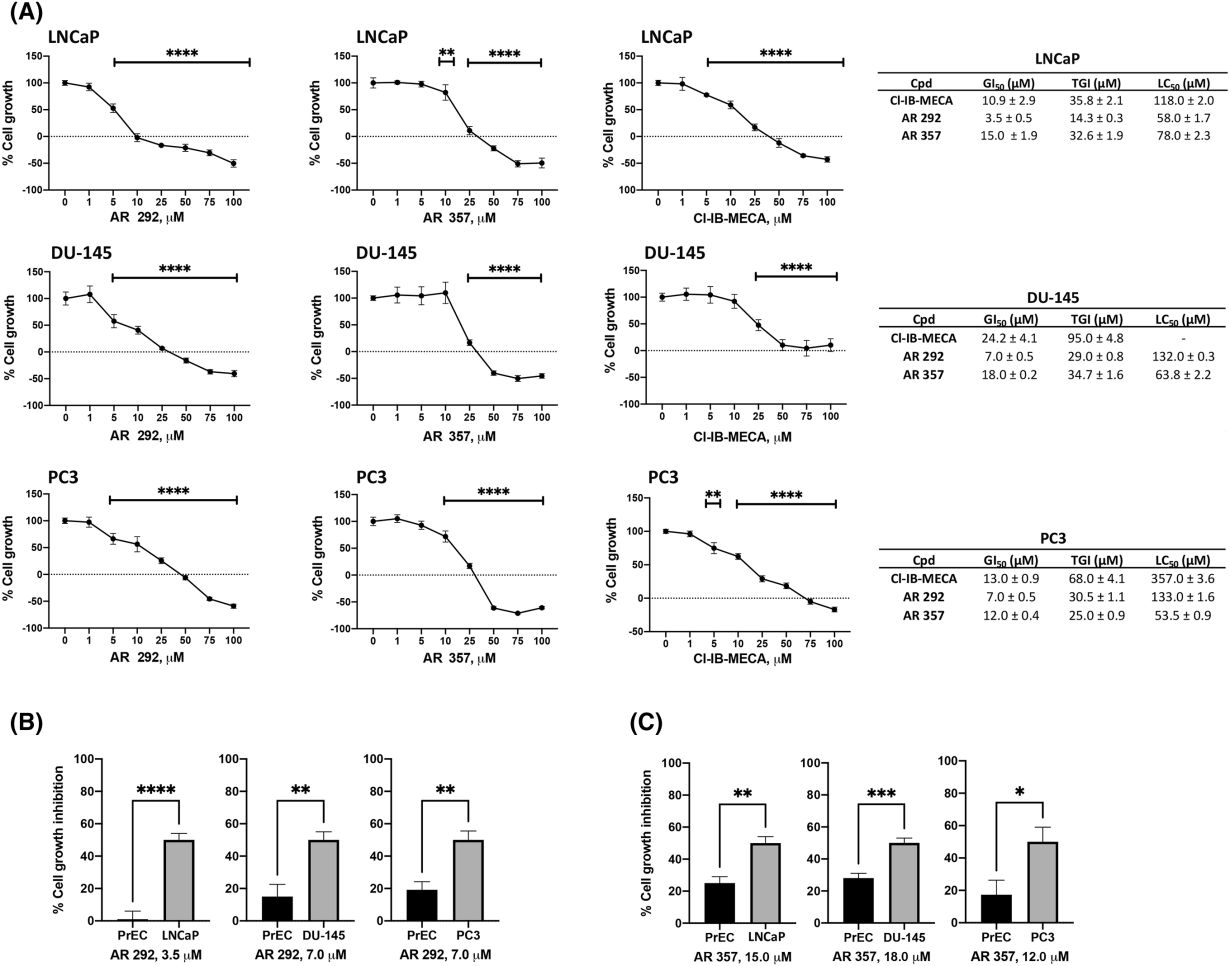
Effects of AR 292 and AR 357 on PCa lines growth. (A) PCa cell growth was evaluated by SRB assay after 48 h of treatments. GI_50_ represents the drug concentration (μm) required to inhibit 50% net of cell growth. Total growth inhibition (TGI) represents the drug concentration (μm) required to inhibit 100% of cell growth. Lethal concentration 50 (LC_50_) represents the drug concentration (μm) required to kill 50% of the initial cell number. Data shown are expressed as mean + SEM of three separate experiments. Data shown are expressed as mean + SEM of three separate experiments. One‐way ANOVA with Tukey's multiple comparison test showed **P < 0.01; ****P < 0.0001. (B, C) PrECs growth was evaluated by MTT assay after 48 h of treatments with AR 292 and AR 357 at the GI_50_ doses specific to each PCa cell line, to compare the effect in cancer and normal cells. Data shown are expressed as mean ± SEM of three separate experiments. One‐way ANOVA with Tukey's multiple comparison test showed *P < 0.05; **P < 0.01; ***P < 0.001; ****P < 0.0001.

AR 292 and AR 357 are A_3_AR ligands, and their cytotoxic effects in PC3 cells were previously demonstrated by Spinaci et al. (Fig. S1) [14]. Thus, the first objective of the current study was to evaluate the effect of various concentrations of these A_3_AR antagonists (1–100 μm) in other human PCa cell lines with characteristics different from PC3, namely LNCaP and DU‐145. Cl‐IB‐MECA, an A_3_AR full agonist with high affinity and very good selectivity, was used as a reference compound. It is an A_3_AR full agonist with high affinity and very good A_3_AR selectivity. As shown in Fig. 1A, A_3_AR ligands were able to decrease PCa cell growth, reaching Growth Inhibition 50 (GI_50_) values of 3.5 μm in LNCaP, 7 μm in DU‐145, and 7 μm in PC3 for AR 292, while for AR 357, LNCaP had a GI_50_ of 15 μm, DU‐145 18 μm and PC3 12 μm. Both compounds showed a better effect than Cl‐IB‐MECA. PrECs have also been used to explore the toxicity of A_3_AR ligands in normal cells. The data displayed an absence of toxicity or lower toxicity in PrECs than in the PCa cell lines used in this study (Fig. 1B,C).

### A_3_AR antagonists induced a proliferation blockade in PCa cells

**Fig. 2:**
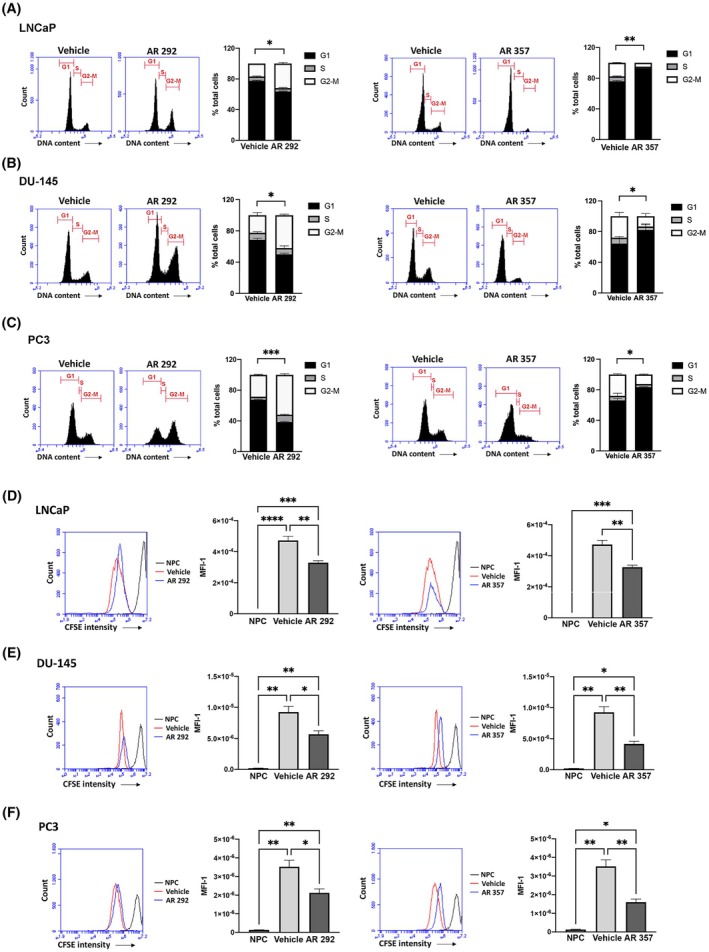
Effects of AR 292 and AR 357 on PCa cell growth. (A–C) Cell cycle analysis in LNCaP (A), DU‐145 (B), and PC3 (C) cells treated with AR 292, AR 357 or with vehicle for 48 h. Histograms are representative of three experiments and the relative numbers represent the percentage of cells in each cell cycle phase. Statistical analysis was calculated using the percentage of cells in each cell cycle phase (mean ± SEM of three experiments). (D–F) cells were stained with carboxyfluorescein succinimidyl ester (CFSE) and then seeded and cultured with AR 292, AR 357 or with vehicle for 48 h. Following this, cells were harvested and the CFSE fluorescence signal was measured. NPC, non‐proliferative control cells. Statistical analysis was calculated using the inverse of CFSE mean fluorescence intensity values (MFI‐1) (mean ± SEM of three experiments). One‐way ANOVA with Tukey's multiple comparison test showed *P < 0.05; **P < 0.01; ***P < 0.001; ****P < 0.0001.

We investigated the effects of A_3_AR antagonists at GI_50_ doses specific to each cell line on cell cycle progression. As shown in Fig. 2A–C, after treatment with AR 292, the accumulation of cells in the G2/M phase was evident in all PCa cell lines (approximately 30% in LNCaP, *P* = 0.0482; 40% in DU‐145, *P* = 0.0133; 50% in PC3, *P* = 0.0003). Instead, when cells were treated with AR 357, the percentage of cells in the G1 phase increased (approximately 90% in LNCaP, *P* = 0.0040; 90% in DU‐145, *P* = 0.0227; 80% in PC3, *P* = 0.0179).

To assess the proliferation rate at the single‐cell level, the cells were stained with CFSE. This stain is evenly distributed in the cytoplasm and stoichiometrically divides into daughter cells during cell division. Data in Fig. 2D–F show that cell proliferation was clearly decreased in cells treated with AR 292 and AR 357 in comparison to non‐proliferative control cells (NPC), with a marked action on DU‐145, followed by PC3 and LNCaP cells. The AR 292 ligand induced a decrease of approximately 30% in LNCaP (*P* = 0.0033), 40% in DU‐145 (*P* = 0.0216), and 40% in PC3 (*P* = 0.0198) cells; AR 357, instead, induced a decrease of approximately 30% in LNCaP (*P* = 0.0031), 55% in DU‐145 (*P* = 0.0066), and 55% in PC3 (*P* = 0.0068) cells. Thus, our data demonstrate that A_3_AR antagonists play a role in PCa cell proliferation.

### A_3_AR antagonist exposure induced PCa cell death

**Fig. 3:**
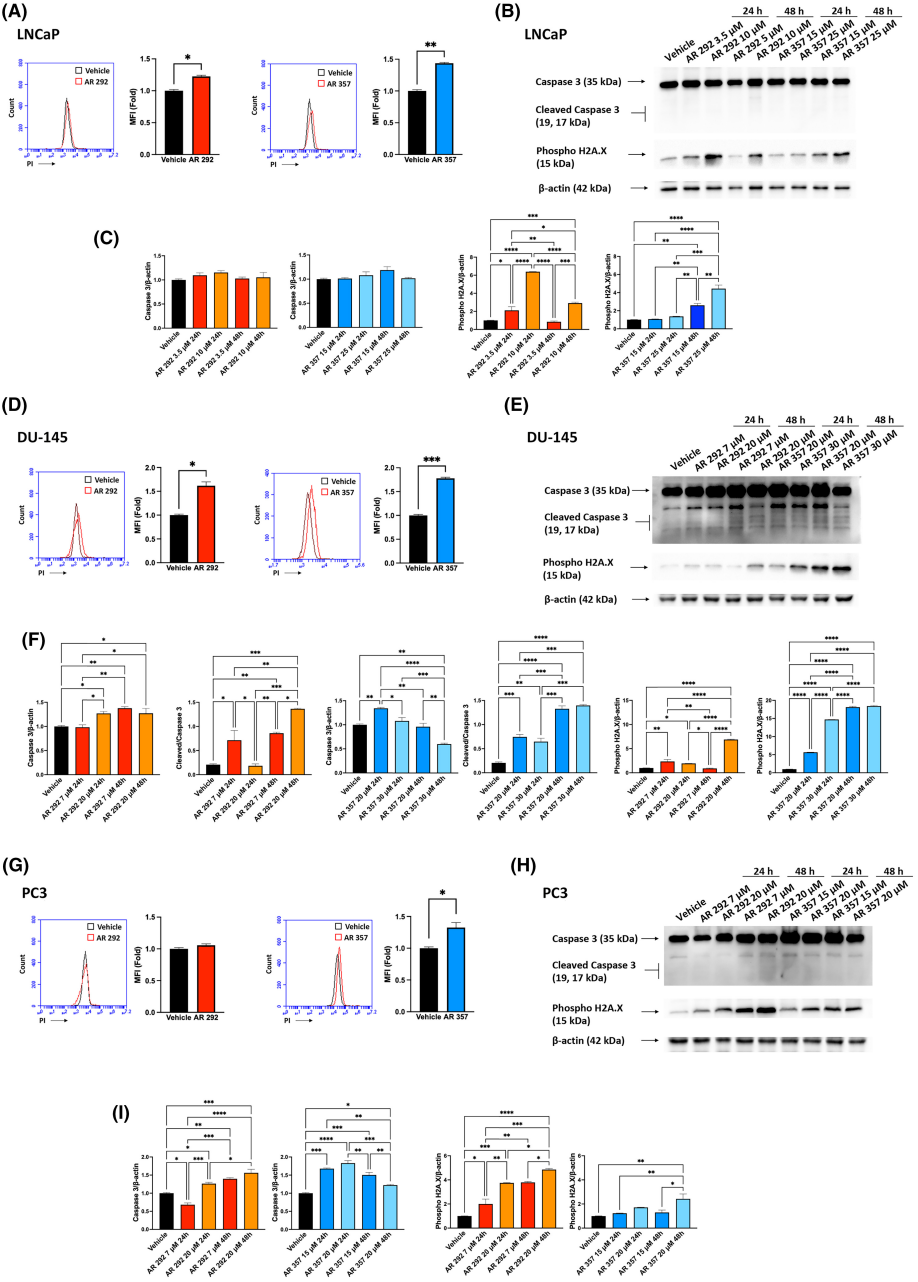
Pca cells were cultured with AR 292 and AR 357 for up to 48 h. (A, D, G) Flow cytometric analysis was performed, followed by propidium iodide (PI) staining. Histograms are representative of one of three separate experiments. For statistical analysis, MFI‐treated cell values were normalized to those of the vehicle. (B, E, H) Western blot analysis of Caspase‐3 and phospho H2A.X in PCa treated as above described. Blots are representative of three experiments. (C, F, I) Cleaved Caspase‐3 protein levels were determined with respect to Caspase‐3 levels. Caspase‐3 and phospho H2A.X densitometry values were normalized to ß‐Actin used as a loading control. Folds (mean ± SEM of three experiments) = changes compared with the vehicle. One‐way ANOVA with Tukey's multiple comparison test showed *P < 0.05; **P < 0.01; ***P < 0.001; ****P < 0.0001.

To explore whether A_3_AR ligand‐induced growth inhibition was associated with cell death, we evaluated the effect of GI_50_ doses of AR 292 and AR 357 in LNCaP, DU‐145, and PC3 cells after 48 h of treatment. Flow cytometry analysis of PI incorporation showed that AR 292 had weak toxic action, resulting in cell death in LNCaP (*P* = 0.0268) and DU‐145 cells (*P* = 0.0103), while compound AR 357 appeared to be effective in all cell lines, especially in DU‐145 cells (LNCaP *P* = 0.0445, DU‐145 *P* = 0.0009, PC3 *P* = 0.0495) (Fig. 3A,D,G). To quantify DNA damage, we evaluated the nucleosomal histone protein H2A.X, specifically phosphorylated (γ‐H2A.X), adjacent to DNA double‐strand breaks. In Fig. 3B,E,H, blots show increased levels of phospho H2A.X after treatment with both A_3_AR antagonists.

To investigate the type of cell death, we evaluated the activation of caspase‐3. As shown in Fig. 3B,E,H, the cleaved form of caspase‐3 was present after AR 292 and AR 357 treatment only in DU‐145 cells. Enhanced Annexin V staining, indicative of apoptotic cell death, was also observed in this cell line (AR292 *P* = 0.0043; AR 357 *P* = 0.0038) (Fig. S2A), whereas no changes were observed in LNCaP and PC3 cells (data not shown). To further confirm the activation of programmed cell death in DU‐145 cells, Bcl‐xL expression and the degradation of PARP‐1 were investigated. The data demonstrated the impairment of the anti‐apoptotic protein Bcl‐xL and the proteolytic cleavage of PARP‐1 after 48 h of treatment with both compounds (Fig. S2B,C).

### Drug transporter gene profile in normal and neoplastic prostate cell lines

**Fig. 4:**
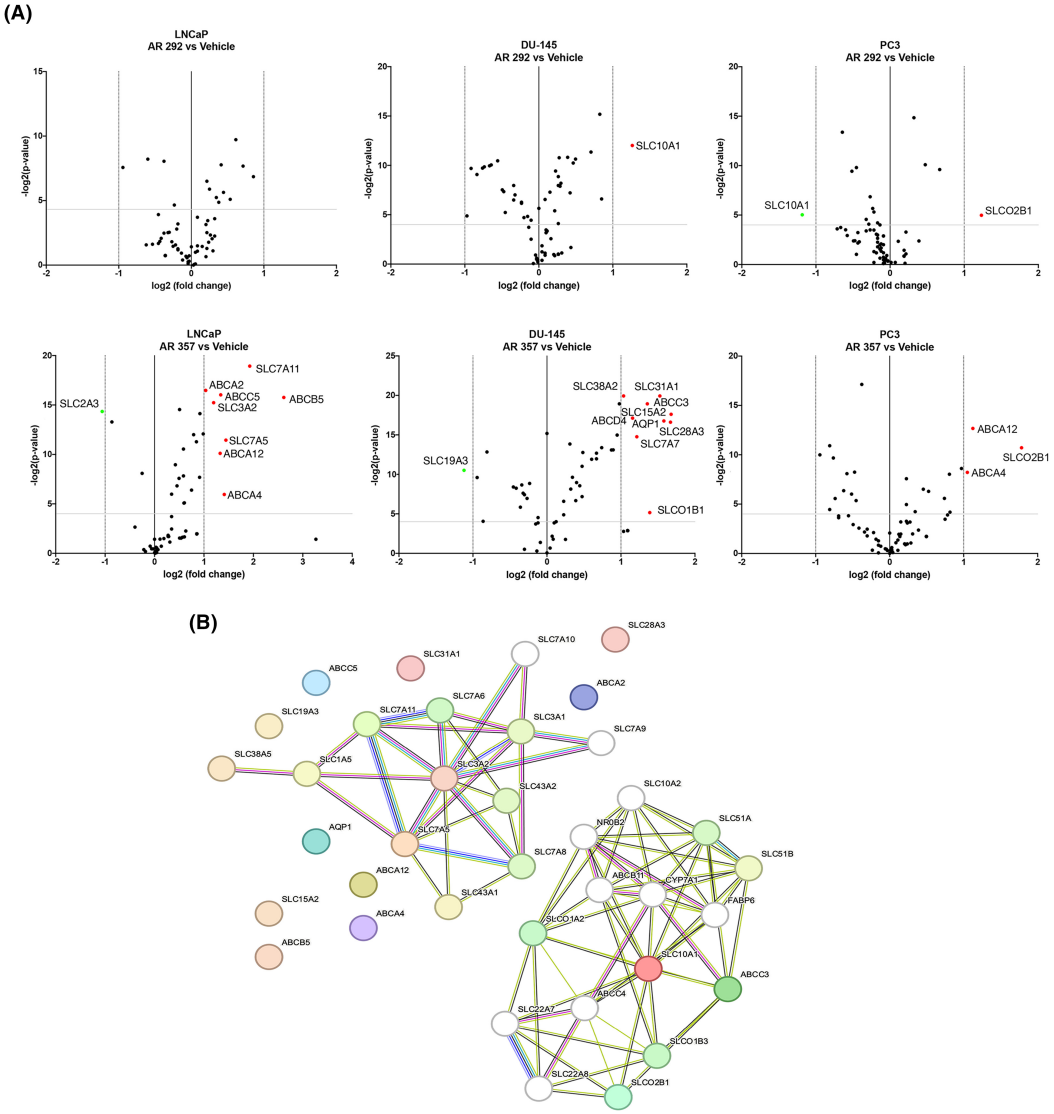
Differential gene expression analysis in PCa cell lines after AR 292 or AR 357 treatment in three biological‐independent experiments. (A) The volcano plot illustrates the relative expression levels for each gene depicted as log 2 (*n*‐fold) plotted against −log_2_ (*P*‐value) between untreated and AR 292 treated cells and untreated or AR 357 treated cells. Horizontal bars represent a significance level of *P* = 0.05; vertical bars represent the fold change threshold (fold difference ≥ 2). The red spots represent upregulated genes, and green spots represent downregulated differentially expressed genes. The black spots indicate non‐differentially expressed genes. (B) The protein–protein interaction network derived from STRING connects the modulated genes (PPI enrichment, *P*‐value <1.0e‐16). One‐way ANOVA with Tukey's multiple comparison test was used.

Drug membrane transporters from the ATP‐binding cassette (ABC) subfamilies A, B, C, and D, solute carrier transporters (SLC), vacuolar H + ‐ATPases, copper pumps, aquaporins (AQP), and voltage‐dependent anion channels play a significant role in drug resistance [17]. Thus, their expression was evaluated in PrECs, as well as in androgen‐sensitive LNCaP and androgen‐resistant PC3 and DU‐145 lines using the RT^2^ Profiler PCR Array. As shown in Tables S1–S4, each cell line showed an individual gene expression profile with respect to PrECs. Thus, we evaluated whether drug transporter expression was modulated by the A3AR antagonist treatment. The analysis revealed that among the modified genes, there was significant modulation of three genes in cells treated with AR 292 and 20 genes in those treated with AR 357, compared to the control (Fig. 4A, Table S4). Most of these genes are functionally associated. Interestingly, six ABC transporter genes, *AQP1*, and the 10 SLC families are related to hypoxic conditions. Among these genes, *ABCA4*, *ABCC5*, *AQP1*, *SLC3A2*, *SLC7A5*, *SLC7A11*, *SLC31A1*, and *SLC38A5* are implicated in the ferroptosis pathway [18]. In addition, the protein–protein interaction (PPIs) network confirmed the functional associations between these selected key genes (Fig. 4B).

### Evidence of ferroptosis in DU‐145 and PC3 cell lines after treatment with A_3_AR antagonists

**Fig. 5:**
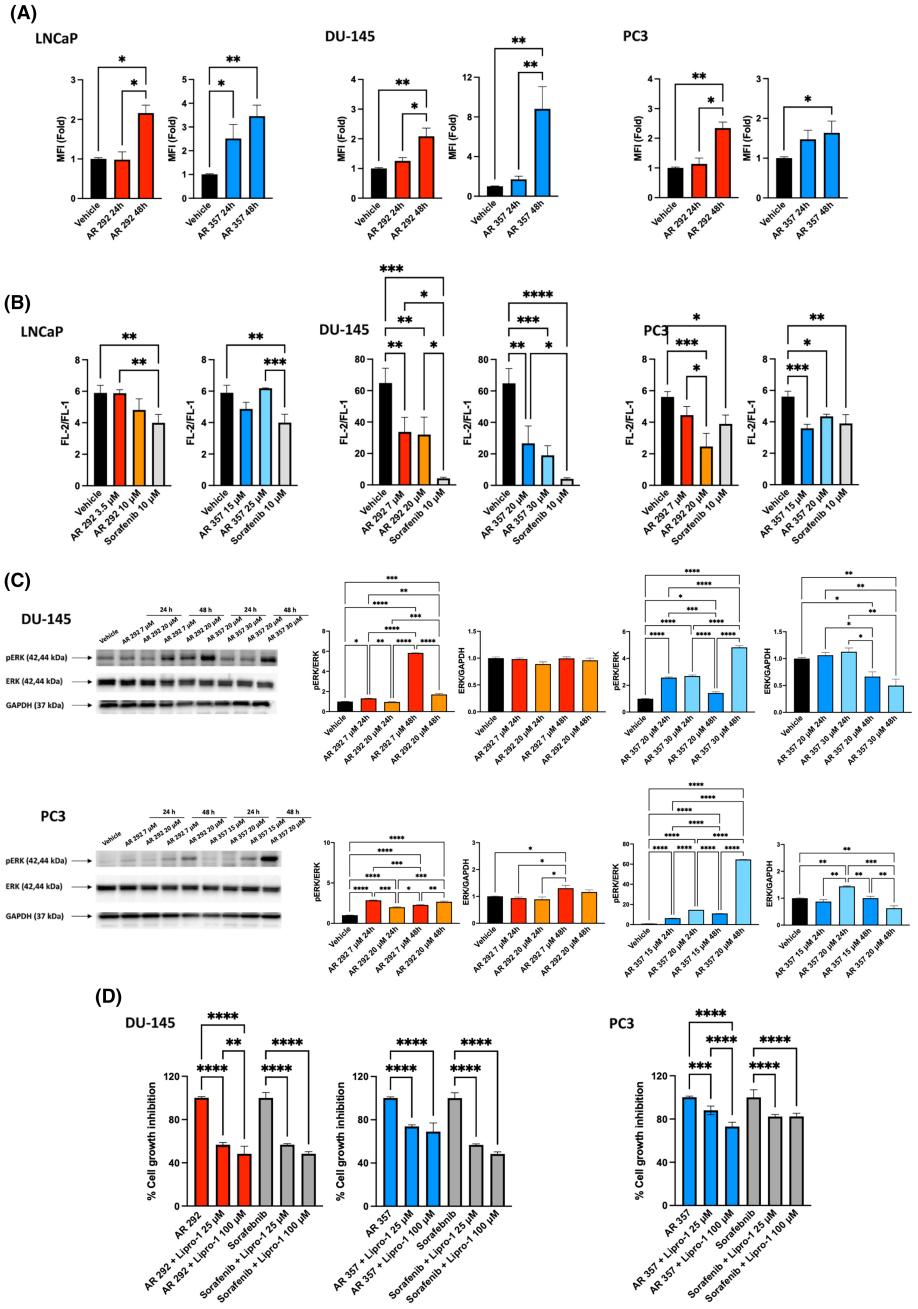
Evidence of ferroptosis cell death. (A) Cells were stained with DCFDA before flow cytometric analysis to evaluate ROS generation. For statistical analysis, values of MFI‐treated cells were normalized to those of the vehicle. (B) Generation of lipid ROS was determined by the C11‐BODIPY (581/591) probe. The ratio of FL‐2 to FL‐1 was determined by cytofluorimeter. For statistical analysis, the mean of MFI + SEM of three experiments was used. (C) Western blot analysis of pERK and ERK in PCa cells cultured with AR 292 and AR 357 for up to 48 h. Blots are representative of three experiments. The pERK protein levels were determined with respect to ERK levels. ERK densitometry values were normalized to GAPDH used as the loading control. Folds (mean ± SEM of three experiments) = changes compared to the vehicle. For statistical analysis, densitometric values related to treated cells were normalized to those of the vehicle. One‐way ANOVA with Tukey's multiple comparison test showed *P < 0.05; **P < 0.01; ***P < 0.001; ****P < 0.0001. (D) PCa cell growth was evaluated by SRB assay after 48 h of treatment with AR 292, AR 357, and Sorafenib alone or in combination with Liproxstatin‐1 (Lipro‐1). Percentages of cell growth inhibition after treatment with AR 292, AR 357, and Sorafenib are compared to 100%. For statistical analysis, data shown are expressed as mean ± SEM of three separate experiments and are normalized to AR 292, AR 357, and Sorafenib.

Given the detected modulation of genes involved in ferroptosis, we analyzed the activation of this form of regulated cell death dependent on lipid ROS. To explore whether A_3_AR antagonists induced ferroptosis, we measured ROS production (Fig. 5A). The data showed that the free radical levels were significantly increased after treatment with both compounds at GI_50_ doses, although AR 357 seemed to be the most effective, achieving a percentage increase in ROS production of, respectively, 3‐fold in LNCaP (*P* = 0.0011), 8‐fold in DU‐145 (*P* = 0.0037), and 1.5‐fold in PC3 (*P* = 0.0422) after 48 h of treatment. Low concentrations of AR 292 and AR 357 had no significant effect on ROS levels.

Moreover, DU‐145 and PC3 cells treated with AR 357 showed a dose‐dependent increase in the level of lipid ROS (DU‐145: AR 357 20 μm *P* = 0.0016; 30 μm *P* = 0.0005; PC3: AR 357 15 μm *P* = 0.0006; 20 μm *P* = 0.0118), as did DU‐145 cells after treatment with AR 292 (DU‐145: AR 297 7 μm *P* = 0.0098; 20 μm *P* = 0.0073), as determined by the C11‐BODIPY (581/591) probe, which showed decreased ratios of red to green fluorescence (Fig. 5B). Sorafenib was used as the positive control. These results suggest that A_3_AR antagonists can trigger ferroptotic cell death by increasing lipid ROS levels only in DU‐145 and PC3 cells. LNCaP cells were more resistant to A_3_AR antagonist‐induced cell death; treated cells had lower levels of necrotic death than those that received the vehicle.

Given that MAPKs play a major role in both proliferation and cell death, including ferroptosis [19], and considering that the truncated thio‐Cl‐IB‐MECA, an A_3_AR antagonist, strongly induces phosphorylation of ERK in T24 human bladder cancer cells [20], we wanted to ascertain whether we also had similar activation in our system. The data in Fig. 5C revealed that the A_3_AR ligands were able to activate ERK. At 24 h, peaking at 48 h, both compounds induced phosphorylation of ERK in DU‐145 and PC3 cells. In parallel, the total ERK levels appeared to decrease after 48 h of treatment. In line with earlier observations, the potent ferroptosis inhibitor Lipro‐1 attenuated the cytotoxic effects of AR 292 and AR 357 in DU‐145 and AR 357 in PC3 cells (DU‐145: AR 292 + Lipro‐1 25 μm *P* < 0.0001; AR 292 + Lipro‐1100 μm *P* < 0.0001; DU‐145: AR 357 + Lipro‐1 25 μm *P* < 0.0001; AR 357 + Lipro‐1100 μm *P* < 0.0001; PC3: AR 357 + Lipro‐1 25 μm *P* = 0.0002; AR 357 + Lipro‐1100 μm *P* < 0.0001) (Fig. 5D) at levels comparable to those achieved in combination with sorafenib, which was used as a positive control.

Taken together, these results indicate that A_3_AR antagonists induce ferroptosis in DU‐145 and PC3 cells.

### A_3_AR ligands regulate the hypoxic response in PCa cell lines

**Fig. 6:**
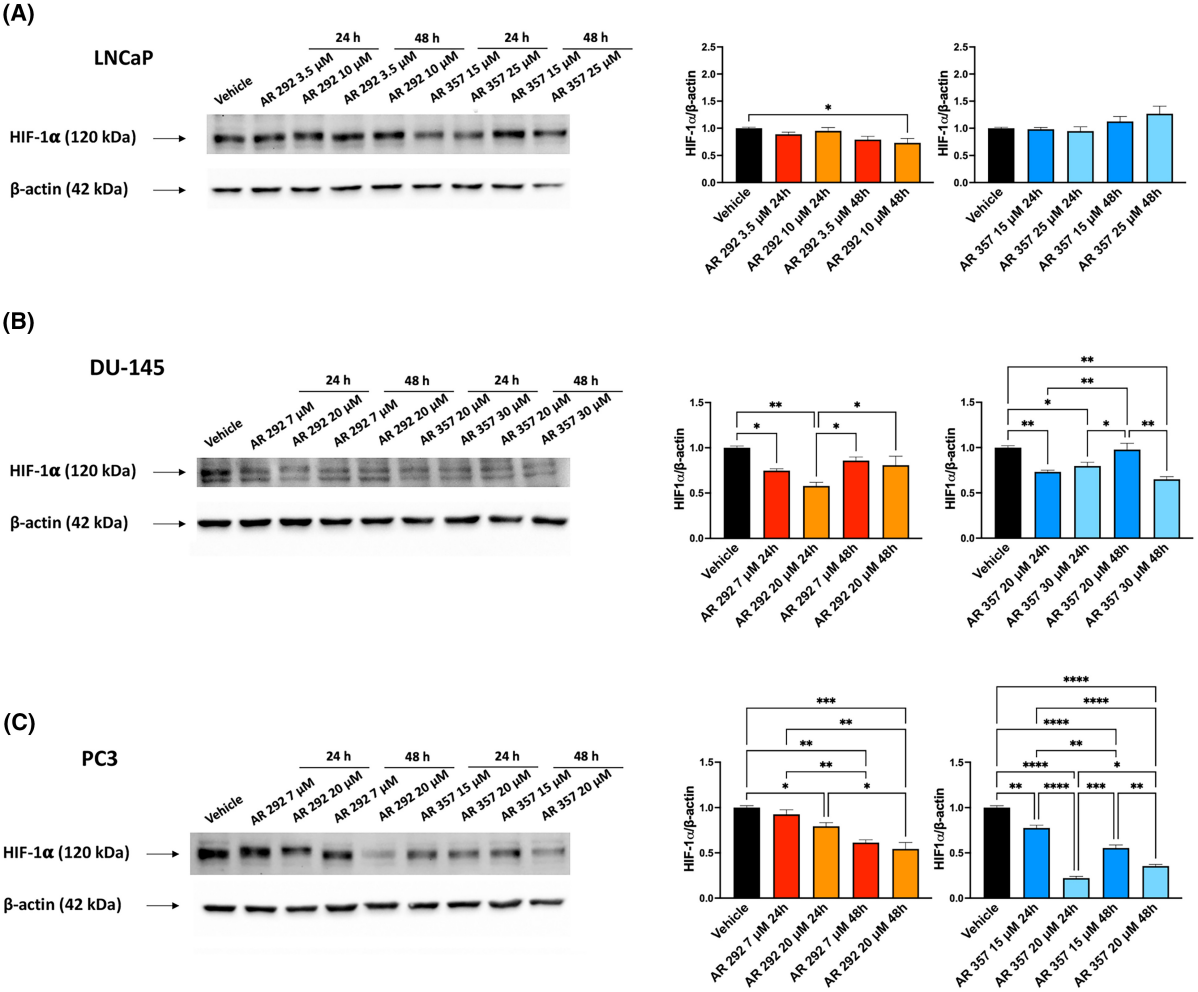
The expression of HIF‐1α after treatment with A_3_AR ligands. Western blot analysis of HIF‐1α in LNCaP (A), DU‐145 (B), and PC3 (C) cells treated with AR 292 and AR 357 for 24 and 48 h. Blots are representative of three experiments. ß‐Actin was used as a loading control. Folds (mean ± SEM of three experiments) = changes compared to vehicle. For statistical analysis, densitometric values related to treated cells were normalized to those of the vehicle. One‐way ANOVA with Tukey's multiple comparison test showed *P < 0.05; **P < 0.01; ***P < 0.001; ****P < 0.0001.

The *SLC19A3* gene modulated in the gene profiler is usually upregulated during hypoxic stress through the activity of HIF1α [21]. This led us to evaluate its levels after treatment with A_3_AR antagonists. Immunoblots showed general impairment in HIF1α protein levels. The decrease was significant but weak in LNCaP cells treated with AR 292 (*P* = 0.0218), while it was more significant in DU‐145 cells, particularly with AR 357 (AR 292 7 μm after 24 h *P* = 0.0027; AR 357 18 μm after 48 h *P* = 0.0018). In PC3 cells treated with both compounds, HIF‐1α protein levels gradually decreased over time (AR 292 7 μm after 48 h *P* = 0.0009; AR 357 12 μm after 24 h *P* = 0.0001) (Fig. 6).

## Discussion

Ado and A_3_ARs play fundamental roles in cancer progression. Thus, A_3_AR ligands have received increased interest for their potential use as therapeutic anti‐tumor drugs. However, their role as anticancer agents is a subject of considerable debate, since both A_3_AR agonists and antagonists can exert anti‐tumor effects. We have previously reported the ability of AR 292 and AR 357 to inhibit the growth of PC3 cells [14]; however, the underlying molecular mechanisms have yet to be elucidated.

In this study, we demonstrated that treatment with the A_3_AR antagonists AR 292 and AR 357 also significantly reduced the growth of androgen‐dependent LNCaP and androgen‐independent DU‐145 cells. Moreover, normal PrECs were less sensitive than PCa cells to AR 292‐ and AR 357‐mediated cytotoxic effects, and this lower toxicity on non‐tumor cells supports their use in therapy.

Both AR 292 and AR 357 reduced PCa cell proliferation. However, AR 292 induced G2/M phase arrest of the cell cycle, whereas AR 357 induced G1 phase arrest. We also determined that both compounds induced DNA damage, as levels of the H2A.X phosphorylated form increased in a time‐dependent manner in all PCa cell lines and also induced cell death. However, the type of cell death process activated depends on the cell type. In particular, compounds AR 292 and AR 357 seem to induce necrotic cell death in LNCaP and PC3 cell lines. Instead, Annexin V positivity, caspase‐3 activation, PARP‐1 degradation, and Bcl‐xL protein impairment, indicative of mitochondrial‐dependent apoptotic cell death, were evidenced in AR 292 and AR 357 treated DU‐145 PCa cells.

Tumor progression, characterized by increased malignancy and reduced survival of cancer patients as a result of increased resistance to chemotherapy and metabolic adaptation to nutrient stress, can be associated with altered expression of drug efflux mediated by ABC, SLC, and APQ drug transporters and ATP‐dependent drug efflux pumps, leading to altered cellular accumulation of anticancer drugs [17]. Focusing on these transporters, we analyzed gene expression in PCa using a drug transporter gene profile that contained most of the transcripts of the ABC, SLC, and APQ families. We compared the expression patterns between non‐tumoral prostate cells and the LNCaP, DU‐145, and PC3 cell lines. We have highlighted that neoplastic transformation of normal epithelial prostate cells is associated with strong modulation of several drug transporter genes. Moreover, each PCa cell line showed an individual gene signature, likely related to the different origins of their metastatic locations (e.g., lymph nodes, bone, and central nervous system metastasis in LNCaP, PC3, and DU‐145 cell lines, respectively), metabolic adaptation to the tumor microenvironment, and chemoresistance genes. Thus, LNCaP cells showed marked and significant downregulation of *TAP‐2*, *ABC‐1 member 1*, *ABCC3/MRP3*, and *MVP* and upregulation of *ABCC1/MRP1* and *ABCC4/MRP4*, and folate, nucleoside, and glucose transporters (*SLC19A1*, *SLC29A2*, *SLC5A4*, respectively), compared to normal PrECs. Compared to normal PrECs, PC3 cells upregulate *VDAC‐2*, *ABC1 member 5*, *ALDH3A1/ALD3*, *ABCB3/MDR5*, *ABCC5/MRP5*, sodium‐coupled nucleoside, copper, and amino acid transporters (*SLC28A2*, *SLC31A1*, and *SLC3A2*, respectively), *SLC25A13/CITRIN*, which stimulate proliferation and invasion, and *ABCF1/GCN20 member 1* by increasing energy production and pyrimidine biosynthesis. Finally, DU‐145 cells showed increased expression of the organic anion transporter (*SLCO2B1*) and reduced sodium‐bile acid and facilitated glucose co‐transporter (*SLC10A1* and *SLC2A3*, respectively) gene expression compared to PrECs. *SLCO4A1* and *SLC7A11* genes were upregulated, whereas *ABCA12*, *SLC38A5*, *SLC5A1*, *SLC28A3*, and *SLCO3A1* were downregulated in all PCa cell lines compared to normal PrECs.

To date, the relationship between the adenosinergic pathway and drug transporters of the ABC and SLC subfamilies has only been partially addressed. Evidence of the direct interaction of A_3_AR agonists at the drug‐binding site(s) of ABCB1 has been reported [22]. ABCB1 overexpression prevents IB‐MECA‐induced cancer cell apoptosis [23]. Cl‐IB‐MECA decreased proliferation, arrested the cell cycle at the G1 phase, and facilitated chemosensitivity by downregulating MDR1 and ABCB1 in pancreatic and hepatocellular carcinoma cell lines [24]. ABCB1 also mediates resistance to the A_3_AR agonist 2‐Cl‐IB‐MECA in human leukemia cells [23]. Moreover, the interaction of A_3_AR ligands with the human multidrug transporter ABCG2 has been reported [25]. Thus, the effect of A_3_AR antagonists on the regulation of drug transporter expression was investigated. *In vitro* treatment of PCa cells with AR 292 and AR 357 altered the gene expression profile of drug transporters. AR 292 induced less change than AR 357, which strongly modified gene expression compared to that of vehicle‐treated PCa cells. From the analysis of pathways, modified genes are involved not only in drug transport and in the regulation of metabolism but also in ferroptosis and in response to hypoxic conditions. In particular, the expression of SLC genes involved in ferroptosis predicts cancer cell prognosis and immunotherapy response [18]. SLC7A11 is a transmembrane protein mainly involved in the anti‐port of cysteine/glutamate on the plasma membrane. It has been demonstrated that its expression is higher in PCa cells than in normal cells [26]. SLC7A11 is a regulator of ferroptosis, with p53 being capable of repressing its expression, thus supporting p53‐mediated tumor growth suppression [26, 27, 28]. Moreover, activation of the HIF‐1α/SLC7A11 pathway exerts anti‐ferroptosis effects [29], and HIF‐1α is the main inducer of hypoxia‐induced ferroptosis resistance [30], as well as an indicator of immunotherapy sensitivity in PCa [31]. Concerning the SLC3 subfamily, the *SLC3A2* gene encodes the CD98hc molecule, which serves as a chaperone for the SLC7A5/LAT1, SLC7A7/x(x) and SLC7A11/xCT transporters [32]. SLC19A3 is a ferroptotic molecule found in muscle‐invasive bladder cancer [33]. It is upregulated during hypoxic stress through the direct binding and activity of HIF‐1α [21]. SLC19A3 was downregulated in AR 357‐treated DU‐145 cells, and this reduced expression may result from the reduced HIF‐1α expression found in A_3_AR‐treated PCa cell lines. The organic anion transporting polypeptide (OATP) family of SLCO transporters has been implicated in PCa disease progression, probably by the transport of hormones or drugs. SLCO transporters with the most recognized role in steroid uptake have been consistently detected in primary PCa [34]. The upregulation of SLCO1B1 induces ferroptosis in colon cancer cells [35]. SLCO2B1, a heme transporter that enhances cellular iron availability [36], mediates the transport of dehydroepiandrosterone (DHEAS), a precursor of testosterone [37]. Thus, changes in SLCO2B1 expression may influence steroid uptake and response to ADT, abiraterone, and docetaxel, which are transported by SLCO2B1 [38]. SLC38A5/SNAT5 solute carriers transport glutamine, methionine, glycine, and serine [39]. It is overexpressed in several cancer types [40] to support the demand for the four amino acids required for the growth, proliferation, and survival of cancer cells [41]. Inhibition of SLC38A5 triggers ferroptosis signaling in pancreatic ductal adenocarcinoma by downregulating GSH levels and GSH‐related genes [42]. Blockage of the expression and function of SLC38A5 induces ferroptosis and oxidative stress in triple‐negative BC cells [39]. Thus, the reduction of its expression in AR 292‐treated LNCaP cells is in agreement with a recent report of *in vitro* and *in vivo* inhibitory effects of *SLC38A5* gene silencing on BC growth [43]. Regarding the ABCA family, overexpression of *ABCA12* was observed in AR 357‐treated LNCaP and PC3 cells. ABCA12 downregulation impairs cancer stemness and chemoresistance [44], and its overexpression appears to be associated with metformin resistance in PCa [45]. Overexpression of ABCA4 is associated with a complete chemotherapy response in advanced stages of ovarian carcinoma [46], which suggests that there is a positive effect on its overexpression in AR‐357‐treated LNCaP and PC3 cells. SLC10A1 is a biomarker for ferroptosis [47]. Overexpression of SLC10A1, which inhibits the production of Ado and decreases HIF‐1α mRNA and protein expression in cancer cells [48], is consistent with the reduced expression of HIF‐1α in AR 292 and AR 357‐treated PCa cell lines.

Based on these profiler results, we first investigated the possible involvement of ferroptosis in PCa cell death. Given that lethal lipid ROS is the driving force of cell death in ferroptosis, we analyzed both ROS production and lipid peroxidation in PCa cell lines. Treatment with A_3_AR ligands increased ROS levels; however, lipid peroxidation was detected only in DU‐145 cells treated with both compounds and in PC3 cells treated with AR 357, suggesting that ferroptosis was induced only in these p53‐mutated cell lines. In addition, ERK1/2 activation, which is involved in ferroptosis induction, was detected in the same cell lines. Similarly, in T24 bladder cancer cells, truncated thio‐Cl‐IB‐MECA, an A_3_AR antagonist, strongly induced ERK phosphorylation‐mediated apoptosis [20]. Finally, the activation of ferroptosis was confirmed by the ability of Lipro‐1, a specific ferroptotic inhibitor, to partially inhibit the cytotoxic effects of A_3_AR ligands.

Solid tumors have developed robust ferroptosis resistance, and HIF‐1α has been shown to be the main driver of this resistance under hypoxic conditions [30]. Since both HIF‐1α and A_3_AR are overexpressed in cancer, the link between A_3_AR stimulation and the modulation of HIF‐1α expression under hypoxic conditions has been explored [49, 50]. A_3_AR stimulation by Ado causes HIF‐1α overexpression in melanoma cell lines in response to hypoxia [49]. In our study, high basal levels of HIF‐1α protein were observed in LNCaP, DU‐145, and PC3 cells, and a time‐dependent decrease was observed in A_3_AR ligand‐treated DU‐145 and PC3 cell lines, but not in the LNCaP cell line. Similarly, the A_3_AR antagonist blocked HIF‐1α protein accumulation in A375 melanoma cells [49]. Collectively, our results suggest cooperation between A_3_AR ligand‐mediated cytotoxic effects and HIF‐1α‐mediated hypoxic effects.

It should be stressed, however, that A_3_AR ligands such as the agonists IB‐MECA and Cl‐IB‐MECA used in the literature as anti‐tumor agents exert cytotoxic effects through A_3_AR‐dependent and A_3_AR‐independent mechanisms. Given that derivatives bearing a 2‐phenethylamino group at the *N* ^6^‐position of Ado, such as AR 292, were found to exert higher A_3_AR affinity and selectivity than the corresponding *N* ^6^‐(2,2‐diphenylethyl) analogs, such as AR 357 [17], and the latter showed the highest anti‐tumor activity, the A_3_AR antagonist‐induced anticancer effects seem to be mediated by both A_3_AR‐dependent and ‐independent mechanisms. AR 292‐ and AR 357‐mediated PCa cytotoxicity seems to be partially A_3_AR‐independent. In contrast, as reported in the literature for the capability of A_3_AR agonists to enhance HIF‐1α mRNA and protein expression in different cancers, our results show a reduction in HIF‐1α protein levels in A_3_AR antagonist‐treated PCa cells, likely supporting an A_3_AR‐dependent effect.

In conclusion, A_3_AR antagonists are capable of blocking proliferation and cell death regardless of the characteristics of the PCa cells analyzed, modulating the sensitivity to the ferroptosis process and the response to hypoxic conditions. Given these promising results, further in‐depth studies are required to evaluate the possibility of using these compounds in therapy.

## Conflict of interest

No conflict of interest is present in any authors with the present data.

## Peer review

The peer review history for this article is available at https://www.webofscience.com/api/gateway/wos/peer‐review/10.1002/2211‐5463.70024.

## Author contributions

Conceptualization, CP, GS, and RV; methodology, MBM and CA; software, MBM and CA; validation, RV, CL, CP, and GS; formal analysis, GS; investigation, MBM; data curation, MBM and CA; writing—original draft preparation, GS; writing—review and editing, MBM, CA, MN, CA, CC, AS, LZ, MG, RV, CL, CP, MS, LS, ML, and VC; visualization, MBM and CA; supervision, GS; project administration, GS; funding acquisition, GS and RV, CP have full access to all the data in the study and take responsibility for the integrity of the data and the accuracy of the data analysis. All the authors have read and agreed to the published version of the manuscript.

## Supporting information


**Fig. S1.** Chemical structures of the A 3 AR ligands Cl‐IB‐MECA, AR 292, and AR 357.

**Fig. S2.** Prostate cancer cells were cultured with AR 292 and AR 357 for up to 48 h.

**Table S1.** Gene expression profile by RT2 profiler PCR array in LNCaP cell lines compared to PrEC cells.
**Table S2.** Gene expression profile by RT2 profiler PCR array in DU‐145 cell lines compared to PrEC cells.
**Table S3.** Gene expression profile by RT2 profiler PCR array in PC3 cell line compared to PrEC cells.
**Table S4.** Gene expression profile by RT2 profiler PCR array in PCa cell lines treated with AR 292 and AR 357.

## Data Availability

The data presented in this study are available upon request from the corresponding author.
