# Supplementary figures and images for: Adenosine A3 receptor antagonists as anti‐tumor treatment in human prostate cancer: an in vitro study
Source: FEBS Open Bio. 2025 Apr 3;15(7):1159–75. doi: 10.1002/2211-5463.70024 (PMC12226426; doi:10.1002/2211-5463.70024)

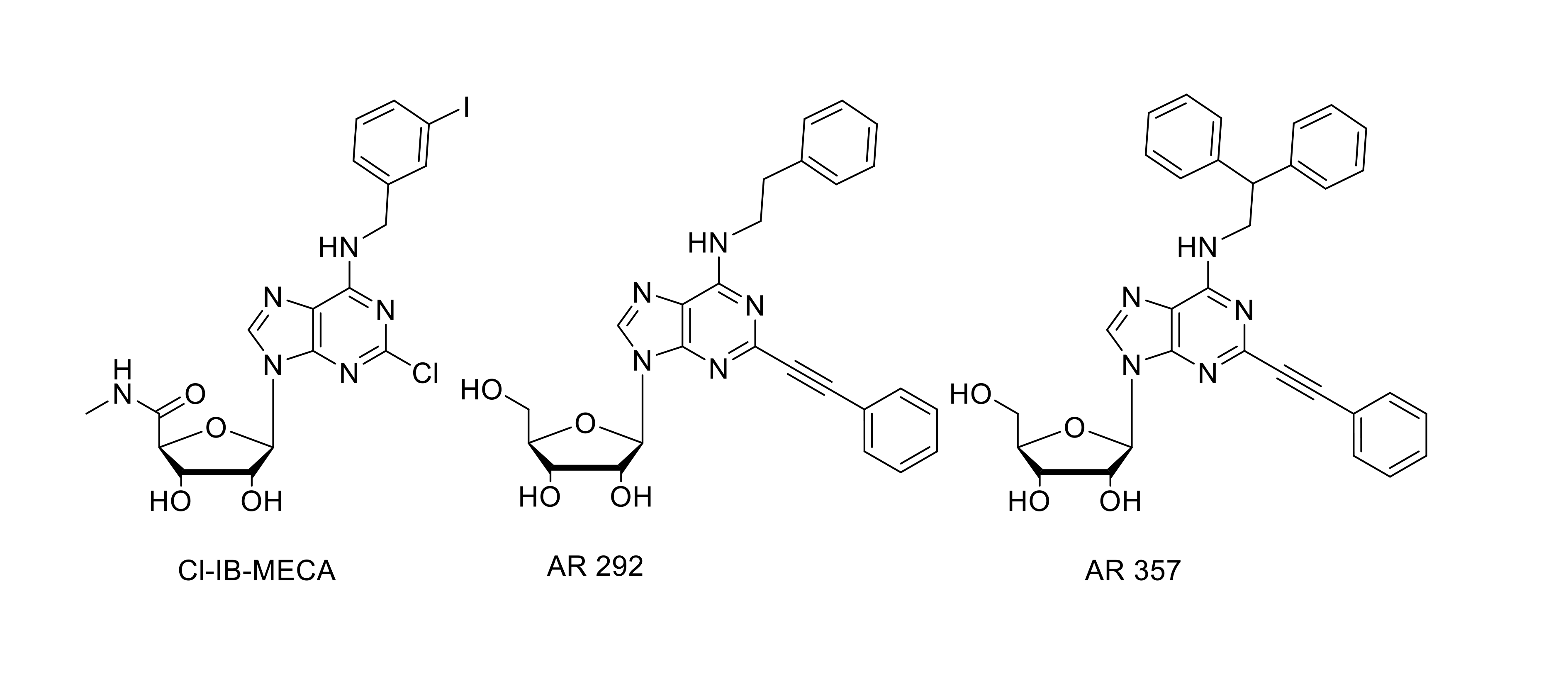

Supplement: Supplementary file 1 — Fig. S1. Chemical structures of the A 3 AR ligands Cl‐IB‐MECA, AR 292, and AR 357. [file FEB4-15-1159-s001.tiff]

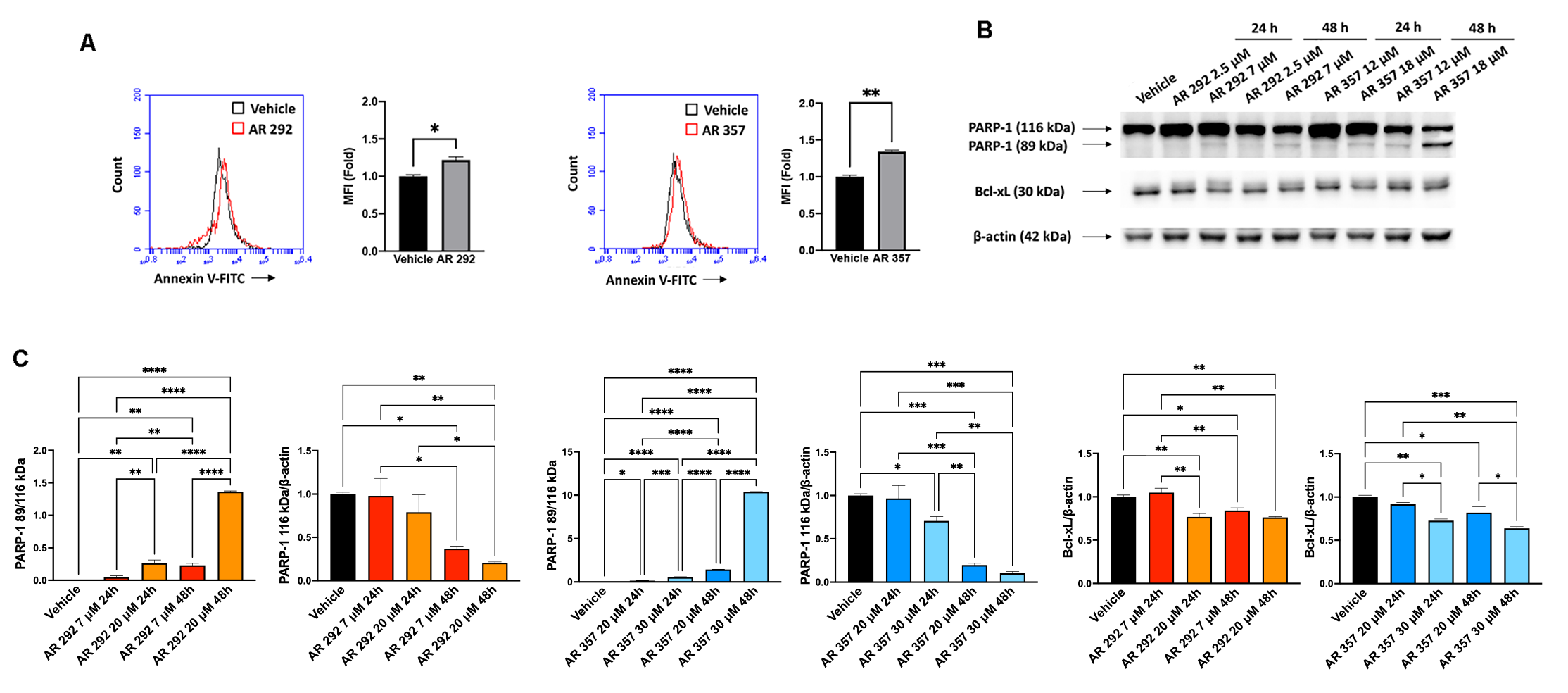

Supplement: Supplementary file 2 — Fig. S2. Prostate cancer cells were cultured with AR 292 and AR 357 for up to 48 h. [file FEB4-15-1159-s003.tiff]
