# Supplementary material for: Adenosine A3 receptor antagonists as anti‐tumor treatment in human prostate cancer: an in vitro study
Source: FEBS Open Bio. 2025 Apr 3;15(7):1159–75. doi: 10.1002/2211-5463.70024 (PMC12226426; doi:10.1002/2211-5463.70024)
Supplement: Supplementary file 3 — Table S1. Gene expression profile by RT2 profiler PCR array in LNCaP cell lines compared to PrEC cells. Table S2. Gene expression profile by RT2 profiler PCR array in DU‐145 cell lines compared to PrEC cells. Table S3. Gene expression profile by RT2 profiler PCR array in PC3 cell line compared to PrEC cells. Table S4. Gene expression profile by RT2 profiler PCR array in PCa cell lines treated with AR 292 and AR 357. [file FEB4-15-1159-s002.docx]

**Table S1.** Gene expression profile by RT2 profiler PCR array in LNCaP cell lines compared to PrEC cells. Genes with > 2-fold difference in mRNA expression and p-value <0.05 in LNCaP cells compared to PrEC were shown.

|  |  | | |  |  |
| --- | --- | --- | --- | --- | --- |
| LNCaP | | | | | |
| Gene Symbol | **Fold** | **p-value** | **Gene Symbol** | **Fold** | **p-value** |
| *ABCA1* | -94.51 | 0.000018 | ***ABCA2*** | 4.98 | 0.000079 |
| *ABCA12* | -4.06 | 0.000353 | ***ABCB11*** | 21.30 | 0.000002 |
| *ABCA4* | -2.36 | 0.001510 | ***ABCB6*** | 5.69 | 0.001026 |
| *ABCC10* | -3.24 | 0.000148 | ***ABCC1*** | 2.15 | 0.034322 |
| *ABCC2* | -2.59 | 0.001094 | ***ABCC4*** | 6.11 | 0.000311 |
| *ABCC3* | -311.20 | 0.000009 | ***ABCD1*** | 19.46 | 0.002937 |
| *ABCG2* | -15.81 | 0.000045 | ***ABCD4*** | 2.09 | 0.000534 |
| *MVP* | -50.11 | 0.000012 | ***AQP1*** | 2.61 | 0.000347 |
| *SLC16A1* | -2.63 | 0.000948 | ***ATP7A*** | 4.12 | 0.000479 |
| *SLC16A2* | -12.59 | 0.000054 | ***ATP7B*** | 5.75 | 0.008606 |
| *SLC16A3* | -105.93 | 0.000014 | ***SLC15A2*** | 2.38 | 0.000415 |
| *SLC19A3* | -3.33 | 0.000551 | ***SLC19A1*** | 2.56 | 0.000006 |
| *SLC28A3* | -19.87 | 0.000062 | ***SLC19A2*** | 3.64 | 0.000559 |
| *SLC2A1* | -9.10 | 0.000001 | ***SLC22A1*** | 5.44 | 0.000364 |
| *SLC38A5* | -237.35 | 0.000005 | ***SLC22A3*** | 14.73 | 0.000000 |
| *SLC3A1* | -2.13 | 0.002911 | ***SLC29A2*** | 3.64 | 0.013283 |
| *SLC5A1* | -2.31 | 0.001631 | ***SLC31A1*** | 2.60 | 0.000002 |
| *SLC7A5* | -55.61 | 0.000001 | ***SLC5A4*** | 7.20 | 0.000066 |
| *SLCO2A1* | -2.15 | 0.006586 | ***SLC7A11*** | 2.96 | 0.000001 |
| *SLCO3A1* | -139.36 | 0.000016 | ***SLCO4A1*** | 2.33 | 0.028471 |
| *TAP1* | -4.97 | 0.000029 |  |  |  |
| *TAP2* | -2.10 | 0.004717 |  |  |  |

**Table S2.** Gene expression profile by RT2 profiler PCR array in DU-145 cell lines compared to PrEC cells. Genes with > 2-fold difference in mRNA expression and p-value <0.05 in DU-145 cells compared to PrEC were shown.

|  |  | | |  |  |
| --- | --- | --- | --- | --- | --- |
| DU-145 | | | | | |
| Gene Symbol | **Fold** | **p-value** | **Gene Symbol** | **Fold** | **p-value** |
| *ABCA1* | -3.54 | 0.000082 | ***ABCA13*** | 9.98 | 0.000125 |
| *ABCA12* | -70.60 | 0.000039 | ***ABCA2*** | 6.16 | 0.000039 |
| *ABCA4* | -4.78 | 0.000413 | ***ABCB6*** | 2.65 | 0.000077 |
| *ATP6V0C* | -2.08 | 0.000048 | ***ABCC2*** | 5.15 | 0.000159 |
| *SLC10A1* | -3.39 | 0.000869 | ***ABCG2*** | 2.14 | 0.000417 |
| *SLC15A2* | -13.17 | 0.000110 | ***ATP7B*** | 6.22 | 0.000106 |
| *SLC16A1* | -4.04 | 0.000003 | ***SLC19A3*** | 3.71 | 0.000263 |
| *SLC16A2* | -25.76 | 0.000045 | ***SLC7A11*** | 2.49 | 0.000090 |
| *SLC22A3* | -2.62 | 0.000169 | ***SLCO1B3*** | 61.75 | 0.000032 |
| *SLC28A3* | -27.78 | 0.000058 | ***SLCO2B1*** | 3.17 | 0.000902 |
| *SLC2A1* | -3.35 | 0.000004 | ***SLCO4A1*** | 4.46 | 0.000031 |
| *SLC2A3* | -5.36 | 0.000166 |  |  |  |
| *SLC38A5* | -549.56 | 0.000005 |  |  |  |
| *SLC5A1* | -4.68 | 0.000428 |  |  |  |
| *SLC7A5* | -2.74 | 0.000006 |  |  |  |
| *SLC7A7* | -2.07 | 0.001607 |  |  |  |
| *SLC7A8* | -628.70 | 0.000009 |  |  |  |
| *SLCO3A1* | -281.88 | 0.000016 |  |  |  |
| *TAP1* | -3.93 | 0.000015 |  |  |  |

**Table S3.** Gene expression profile by RT2 profiler PCR array in PC3 cell line compared to PrEC cells. Genes with > 2-fold difference in mRNA expression and p-value <0.05 in PC3 cells compared to PrEC were shown.

|  |  | | | |  |  |
| --- | --- | --- | --- | --- | --- | --- |
| PC3 | | | | | | |
| Gene Symbol | **Fold** | **p-value** | **Gene Symbol** | **Fold** | | **p-value** |
| *ABCA12* | -5.30 | 0.000098 | ***ABCA13*** | 126.15 | | **0.006320** |
| *ABCG2* | -23.40 | 0.000040 | ***ABCA4*** | 2.40 | | 0.002401 |
| *SLC15A2* | -2.41 | 0.000673 | ***ABCA5*** | 8.33 | | 0.001704 |
| *SLC19A3* | -2.65 | 0.001127 | ***ABCB11*** | 20.50 | | 0.000000 |
| *SLC22A3* | -3.44 | 0.036428 | ***ABCB5*** | 10.73 | | 0.003501 |
| *SLC28A3* | -6.48 | 0.000097 | ***ABCC10*** | 2.02 | | 0.000114 |
| *SLC38A5* | -57.17 | 0.000005 | ***ABCC2*** | 10.93 | | 0.000003 |
| *SLC5A1* | -2.20 | 0.002425 | ***ABCC5*** | 2.05 | | 0.000479 |
| *SLC7A8* | -3.33 | 0.000045 | ***ABCD1*** | 19.77 | | 0.000036 |
| *SLCO2A1* | -10.19 | 0.000107 | ***ABCD3*** | 2.63 | | 0.000193 |
| *SLCO3A1* | -3.42 | 0.000100 | ***ABCD4*** | 5.22 | | 0.000005 |
|  |  |  | ***ABCF1*** | 2.72 | | 0.000174 |
|  |  |  | ***AQP1*** | 2.34 | | 0.017983 |
|  |  |  | ***ATP6V0C*** | 2.35 | | 0.000021 |
|  |  |  | ***ATP7A*** | 4.84 | | 0.000006 |
|  |  |  | ***ATP7B*** | 11.95 | | 0.003339 |
|  |  |  | ***SLC16A2*** | 2.60 | | 0.013603 |
|  |  |  | ***SLC16A3*** | 6.06 | | 0.004657 |
|  |  |  | ***SLC19A2*** | 4.25 | | 0.000450 |
|  |  |  | ***SLC22A1*** | 4.25 | | 0.006541 |
|  |  |  | ***SLC25A13*** | 2.21 | | 0.000407 |
|  |  |  | ***SLC28A2*** | 2.09 | | 0.000672 |
|  |  |  | ***SLC3A1*** | **2.16** | | **0.000165** |
|  |  |  | ***SLC3A2*** | 6.01 | | 0.000310 |
|  |  |  | ***SLC7A11*** | 18.52 | | 0.002965 |
|  |  |  | ***SLC7A7*** | 3.74 | | 0.000437 |
|  |  |  | ***SLCO1B3*** | 391.28 | | 0.002443 |
|  |  |  | ***SLCO4A1*** | 45.12 | | 0.000002 |
|  |  |  | ***VDAC2*** | 6.85 | | 0.000003 |

**Table S4.** Gene expression profile by RT2 profiler PCR array in PCa cell lines treated with AR 292 and AR 357. Genes with > 2-fold difference in mRNA expression and p-value <0.05 in AR 292 or AR 357 treated cells compared to untreated ones were shown.

|  | AR 292 | | | | | | AR 357 | | | | | |
| --- | --- | --- | --- | --- | --- | --- | --- | --- | --- | --- | --- | --- |
|  | **LNCaP** | | **DU-145** | | **PC3** | | **LNCaP** | | **DU-145** | | **PC3** | |
|  | **Fold** | **p-value** | **Fold** | **p-value** | **Fold** | **p-value** | **Fold** | **p-value** | **Fold** | **p-value** | **Fold** | **p-value** |
| *ABCA2* |  |  |  |  |  |  | 2.24 | 0.000019 |  |  |  |  |
| *ABCA4* |  |  |  |  |  |  | 3.43 | 0.000191 |  |  | 2.04 | 0.001182 |
| *ABCA12* |  |  |  |  |  |  | 2.74 | 0.000331 |  |  | 2.14 | 0.000693 |
| *ABCB5* |  |  |  |  |  |  | 8.39 | 0.000000 |  |  |  |  |
| *ABCC3* |  |  |  |  |  |  |  |  | 2.56 | 0.000002 |  |  |
| *ABCC5* |  |  |  |  |  |  | 2.76 | 0.000017 |  |  |  |  |
| *AQP1* |  |  |  |  |  |  |  |  | 2.99 | 0.000009 |  |  |
| *SLC28A3* |  |  |  |  |  |  |  |  | 3.18 | 0.000010 |  |  |
| *SLC3A2* |  |  |  |  |  |  | 2.50 | 0.000024 | 4.06 | 0.000000 |  |  |
| *SLC7A5* |  |  |  |  |  |  | 2.97 | 0.000139 |  |  |  |  |
| *SLC7A11* |  |  |  |  |  |  | 4.14 | 0.000009 | 3.26 | 0.000000 |  |  |
| *SLC10A1* |  |  | 2.40 | 0.000242 | -2.89 | 0.002073 |  |  | 5.39 | 0.000003 |  |  |
| *SLC15A2* |  |  |  |  |  |  |  |  | 3.20 | 0.000005 |  |  |
| *SLC19A3* |  |  |  |  |  |  |  |  | -2.19 | 0.000685 |  |  |
| *SLC31A1* |  |  |  |  |  |  | 2.16 | 0.000355 | 2.88 | 0.000001 |  |  |
| *SLC38A5* | -2.10 | 0.001081 |  |  |  |  |  |  | 4.05 | 0.000002 |  |  |
| *SLCO2B1* |  |  |  |  | 2.23 | 0.014180 |  |  | 2.62 | 0.028022 | 3.38 | 0.000122 |
